# Lessons Learned from Emergency Response Vaccination Efforts for Cholera, Typhoid, Yellow Fever, and Ebola

**DOI:** 10.3201/eid2313.170550

**Published:** 2017-12

**Authors:** Jenny A. Walldorf, Kashmira A. Date, Nandini Sreenivasan, Jennifer B. Harris, Terri B. Hyde

**Affiliations:** Centers for Disease Control and Prevention, Atlanta, Georgia, USA

**Keywords:** vaccination, capacity, emergencies, emergency responses, cholera, Ebola virus disease, typhoid, yellow fever, vaccine-preventable diseases, disease burden, global health, global health security, bacteria, viruses, Ebola virus, Vibrio cholerae, yellow fever virus, Salmonella enterica serovar Typhi, Ebola

## Abstract

Countries must be prepared to respond to public health threats associated with emergencies, such as natural disasters, sociopolitical conflicts, or uncontrolled disease outbreaks. Rapid vaccination of populations vulnerable to epidemic-prone vaccine-preventable diseases is a major component of emergency response. Emergency vaccination planning presents challenges, including how to predict resource needs, expand vaccine availability during global shortages, and address regulatory barriers to deliver new products. The US Centers for Disease Control and Prevention supports countries to plan, implement, and evaluate emergency vaccination response. We describe work of the Centers for Disease Control and Prevention in collaboration with global partners to support emergency vaccination against cholera, typhoid, yellow fever, and Ebola, diseases for which a new vaccine or vaccine formulation has played a major role in response. Lessons learned will help countries prepare for future emergencies. Integration of vaccination with emergency response augments global health security through reducing disease burden, saving lives, and preventing spread across international borders.

In emergency settings, countries must be prepared to respond to public health threats. Prompt vaccine delivery can be a major component of emergency response, especially for populations vulnerable to epidemic-prone, vaccine-preventable diseases (VPDs). Public health emergencies might be triggered by natural disaster; humanitarian emergency; a disease pandemic leading to health systems breakdown, as in the recent Ebola epidemic in West Africa in 2014; or by a specific VPD outbreak not contained by ongoing immunization services. During emergencies affecting health systems in general, vaccination services are frequently disrupted. Emergency vaccination campaigns aim to control VPD outbreaks, reducing the possibility of international spread and thereby enhancing global health security.

For countries to respond rapidly in emergency situations, planning for appropriate and effective vaccine delivery to at-risk populations is essential. The decision to engage in a vaccination response depends on several factors including the risk for a VPD in the emergency situation, characteristics and availability of vaccines for response, and prioritization of vaccination in relation to other public health interventions (*1*). Once a decision is made for a vaccination response, additional issues need to be addressed, including regulatory barriers for unlicensed products, vaccine supply and stockpile access, appropriate cold chain capacity, and designation of roles and responsibilities based on in-country capacity and global partner involvement. Key responsibilities include overall emergency management, coordination of vaccination response, communications and social mobilization, monitoring and evaluation of vaccine implementation, and enhancement of surveillance for adverse events after immunization. To enable countries to respond rapidly to future emergencies, clearly outlining the command structure beforehand for these key responsibilities is essential.

Evaluation of emergency vaccination activities is a major component of the overall response efforts, necessary to refine ongoing activities and document lessons learned. Implementation research can close evidence gaps between licensure and programmatic use of new vaccines (*2*). Emergency vaccination has been well-described and evaluated for outbreak-prone VPDs such as polio, measles, meningitis, yellow fever, cholera, and hepatitis A (*3*). However, as new vaccines, new formulations, or new routes of administration for existing vaccines are developed, licensed, and prequalified by the World Health Organization (WHO), vaccination strategies must be evaluated and reevaluated to ensure the greatest effect for protecting vulnerable populations and preventing spread of disease.

The Global Immunization Division in the Center for Global Health at the Centers for Disease Control and Prevention (CDC) is committed to supporting countries in emergency vaccine delivery planning, implementation, and evaluation in collaboration with other CDC divisions and with international partners. In this report, we highlight the work CDC has conducted to generate evidence that will shape future outbreak response vaccination strategies by using lessons learned specifically from cholera, typhoid, yellow fever, and Ebola. For these diseases, a new vaccine or new vaccine formulation has played a major role in emergency response. Lessons learned will help countries prepare for future emergency outbreak response and contribute to the broader goal of more rapidly containing public health emergencies caused by VPDs, thereby enhancing global health security.

## Cholera

Cholera is an acute diarrheal infection caused by ingestion of toxigenic serogroups O1 and O139 of the bacterium *Vibrio cholerae*. The global burden of cholera is estimated to be 2.9 million cases and 95,000 deaths annually; most cases are reported to WHO from sub-Saharan Africa (*4*). More recently, since 2010, Haiti has made a major contribution to the global burden. Although cholera prevention and control measures have traditionally focused on cholera treatment and improving access to safe water, sanitation, and hygiene (WaSH), oral cholera vaccines (OCVs) have gained prominence as a major complementary tool in comprehensive cholera prevention and control. In 2010, WHO recommended the use of existing OCVs, preemptively in cholera-endemic settings to target high-risk areas or populations or reactively as part of outbreak response activities (*5*). In June 2013, WHO established a global OCV stockpile with an initial stock of 2 million doses with funding from multiple partners (*6*). In November 2013, the Global Alliance for Vaccines and Immunization endorsed funding support for the stockpile for 2014–2018 (*7*). As of March 2017, a total of 41 OCV campaigns have been conducted in 14 countries with vaccine from the global stockpile (*8*).

Three inactivated, whole-cell OCVs are prequalified by WHO and available for global use: Dukoral (killed whole-cell monovalent [O1] cholera vaccine with cholera toxin B subunit; Valneva, Lyon, France); Shanchol (modified killed bivalent [O1 and O139] whole-cell–only cholera vaccines; (Shantha Biotechnics, Hyderabad, India); and Euvichol (modified killed bivalent [O1 and O139] whole-cell–only cholera vaccines; Eubiologics, Seoul, South Korea) (*9*). Two of these vaccines, Shanchol and Euvichol, are available through the global stockpile. Shanchol and Euvichol are recommended for persons >1 years of age, including pregnant women (*10*), in a 2-dose schedule given >14 days apart. Both OCVs are safe, efficacious, and effective in multiple settings (*5**,**11**,**12*). Recently, a single dose of Shanchol showed an efficacy of 63% against severely dehydrating cholera in the short term (6 months), which has major implications for outbreak control (*13*).

CDC has conducted several evaluations of OCV use in emergency settings. In 2010, CDC collaborated with partners including WHO, the Pan American Health Organization, and others to review current evidence for OCV use in emergency settings and conduct real-time modeling to estimate the effect of using a limited supply of available OCV doses during a cholera outbreak in Haiti after the 2012 earthquake (*14*). In the postemergency period in Haiti, CDC conducted additional evaluations of the cholera response to inform future vaccination campaigns. These evaluations included OCV coverage surveys and precampaign and postcampaign knowledge, attitude, and practice (KAP) surveys (*15**,**16*). A postcampaign KAP survey showed an increase in availability of soap and handwashing stations but a decrease in reported treatment of drinking water, highlighting the need for comprehensive communication messages for cholera control during and, if feasible, after OCV campaigns.

In 2013, CDC supported the Thailand Ministry of Public Health in implementing and evaluating a preemptive 2-dose OCV campaign in a refugee camp. Coverage and precampaign and postcampaign KAP surveys showed a high degree of acceptability of the campaign, as well as improvements in WaSH behaviors (*17**,**18*). In 2015, a 2-dose OCV campaign was conducted in Iraq in response to a cholera outbreak affecting ≈255,000 persons living in selected refugee camps, internally displaced persons camps, and collective centers. After the campaign, CDC conducted a coverage survey in collaboration with WHO and the Iraq Ministry of Health; overall, 2-dose coverage was 87%, and 55% of respondents reported receiving other cholera prevention messages (*19*). This evaluation demonstrated the feasibility of successfully implementing an OCV vaccination campaign in a conflict setting as part of an integrated approach to cholera control.

Most recently, 1 million doses of Euvichol were released for use in hurricane-affected departments in Haiti after Hurricane Matthew in October 2016, the first release of Euvichol from the global stockpile. Approximately 830,000 persons in 18 communes were targeted for vaccination with a single dose, the largest single-dose campaign as well as the largest emergency stockpile release to date. CDC was part of the Haiti OCV taskforce that led monitoring and evaluation of the campaign. This effort included improving laboratory capacity for stool cultures and planning for a coverage survey and a single-dose effectiveness study in collaboration with the Ministry of Public Health and Population and other partners. Because of delays, the evaluations were not conducted; however, in-country staff were trained on field survey techniques and laboratory methods for evaluation, which increased their capacity for future evaluation activities.

Athough OCV campaigns have clearly been demonstrated as feasible in emergency and cholera-endemic settings, additional evaluations will address evidence gaps. These gaps include single-dose effectiveness, effect of OCV (1 and 2 doses) on halting an outbreak and reducing disease burden, the effectiveness of a second dose in the setting of a prolonged dosing interval, and how to optimize the integration of OCV and WaSH for both short-term and longer-term cholera control in emergency and cholera-endemic settings. A single OCV dose might be particularly useful in emergency settings and might enable vaccination of a larger population in a shorter timeframe when a 2-dose delivery is challenging.

## Typhoid

Typhoid (typhoid fever), which is caused by the bacterium *Salmonella enterica* serovar Typhi, is responsible for ≈11 million illnesses and 129,000 deaths globally each year (*20*). As is true for cholera, typhoid primarily occurs in southern Asia, sub-Saharan Africa, and parts of the Middle East and Latin America, where limited access to safe water, inadequate sanitation infrastructure, and poor hygiene practices, often as a result of rapid urbanization, favor transmission. Although most disease is endemic, these same factors give typhoid a high epidemic potential, and outbreaks occur periodically, including outbreaks caused by antimicrobial drug−resistant strains (*21**–**24*). Although most typhoid prevention and control efforts have focused on primary measures of WaSH, vaccines are a major complementary strategy. In 2008, WHO recommended use of existing typhoid vaccines for endemic disease control and outbreak control (*25*). More recently, however, a rapid global increase in antimicrobial drug resistance (*26**,**27*) has emphasized the need for more prompt, short-term prevention and control efforts using existing and newer-generation typhoid vaccines.

Two typhoid vaccines have been available for use in several countries since the 1990s. The first vaccine is a single-dose injectable polysaccharide vaccine based on the purified Typhi Vi antigen (ViPS vaccine), which is for use in persons ≥2 years of age. The second vaccine is a multidose, live attenuated, oral Ty21a vaccine available as a capsule formulation for persons ≥5 years of age. Both vaccines are safe, efficacious, and effective in multiple settings. A recently available newer-generation, single-dose, injectable typhoid conjugate vaccine (TCV) has several advantages over current polysaccharide vaccines, including a higher level of vaccine effectiveness, a longer duration of protection, an added booster response, and approval for use in children <2 years of age. Detailed information on the various vaccines is available elsewhere (*25**,**28**,**29*). Despite the large body of evidence and availability of the current typhoid vaccines, vaccine adoption and use has been limited globally.

CDC has been working with partners to plan, monitor, and evaluate emergency use of typhoid vaccine. In 2010, after a category 4 tropical cyclone in Fiji, the Ministry of Health of Fiji conducted an emergency typhoid vaccination campaign with the ViPS vaccine that targeted cyclone-affected areas as part of the postdisaster response. A small proportion of vaccine was also used in an area not affected by the cyclone but that had experienced a typhoid outbreak during the same period. CDC conducted an impact evaluation in collaboration with partners that showed reduction of disease burden in areas where a large proportion of the population was vaccinated compared with unvaccinated areas (*30*).

In a protracted outbreak in Kasese District in Uganda during 2008−2011, CDC coordinated discussions with multiple partners, including the Coalition Against Typhoid, the Uganda Ministry of Health Expanded Program on Immunization, and Sanofi Pasteur (Lyon, France), regarding vaccine use for outbreak control. CDC investigated the protracted nature of the outbreak (*31*) and conducted a cost-effectiveness modeling exercise to support the need for emergency vaccination (*32*). However, a global vaccine shortage caused by a recall on certain lots of the Sanofi ViPS vaccine precluded vaccine use.

CDC is working with WHO in India; Stanford University (Stanford, CA, USA); local hospitals in Navi Mumbai, India; and the Municipal Corporation (local government body) to evaluate the planned introduction of TCV in a public sector program targeting ≈400,000 children 9 months–14 years of age. Although vaccine introduction will occur in a disease-endemic setting, evaluation findings, including safety, effectiveness, acceptability, and impact will provide information for future targeting and use of TCV in emergency settings.

## Yellow Fever

Yellow fever is a viral hemorrhagic fever caused by the yellow fever virus (genus *Flavivirus*), which is transmitted by *Haemagogus* and *Aedes* spp. mosquitoes. Yellow fever is endemic to tropical regions of 47 countries in Africa and South and Central America; >90% of cases and deaths are in Africa (*33*). The number of reported cases is believed to be greatly underestimated because of challenges in surveillance and diagnosis. Yellow fever caused ≈51,000–380,000 severe cases and 19,000–180,000 deaths in Africa in 2013 (*34*).

Current yellow fever vaccines are live attenuated vaccines manufactured from 2 substrains of the 17D strain. A standard 0.5-mL dose is highly efficacious; ≈97.5% of recipients showed development of protective levels of antibodies (*35*) and life-long protection. Many disease-endemic countries have introduced yellow fever vaccine into their childhood immunization schedules since the late 1990s, with or without a preventive mass vaccination campaign for all ages near the time of introduction. However, there are huge gaps in population immunity because some countries have not introduced yellow fever vaccine, coverage in routine immunization programs of many countries is suboptimal, and most adults in countries that did not conduct mass preventive campaigns are unprotected. Furthermore, recent changes in environmental and agricultural conditions have contributed to a worldwide resurgence in the *Ae. aegypti* mosquito, the primary vector in urban settings (*33*). Large urban outbreaks can occur when infected persons move to densely populated urban settings in which population immunity is low and *Ae. aegypti* mosquitoes are present (*33*).

Because outbreak response needs are difficult to predict, a global stockpile of yellow fever vaccine has been maintained since 2001; >90 million doses have been distributed (*36*). The 6 million–dose stockpile had to be replenished multiple times in 2016 because of outbreak response vaccinations in Angola and the Democratic Republic of the Congo (DRC), which led to the use of almost 30 million doses of vaccine (*37*). During the response, a large-scale campaign targeted 8 million persons in Kinshasa, the capital of the DRC, in August 2016. At that time, however, an insufficient vaccine supply was available globally. Fractional-dose yellow fever vaccine administered by subcutaneous and intramuscular injections was evaluated in 2 small, controlled studies in healthy adults (*38**,**39*), but its use in a mass campaign had never been evaluated. With guidance from WHO, the DRC decided to administer a fractional (1/5; 0.1 mL) dose of yellow fever vaccine to all nonpregnant adults and children >2 years of age. Pregnant women and children 9 months−2 years of age received the full dose.

To evaluate whether the immunogenic response observed in persons vaccinated during the mass campaign was sufficient to confer protection against yellow fever virus, CDC partnered with the US Agency for International Development and the Institut Nationale de Recherche Biologique (Kinshasa), the national reference laboratory in the DRC, to conduct a cohort study of 760 persons eligible for vaccination during the campaign. Participants provided blood samples before and 28 days after vaccination; another sample will be collected 1 year after vaccination. If the fractional dose is found to induce a sufficient immune response to confer protection, this result would provide supporting evidence for fractional-dose yellow fever vaccination as a strategy to control outbreaks of yellow fever.

The evaluation in the DRC will provide immunogenicity data on adults and children >2 years of age. However, this evaluation will not provide immunogenicity data for fractional-dose vaccination in children <2 years of age. CDC has partnered with the Uganda Viral Research Institute (Entebbe, Uganda) and the Infectious Diseases Institute of Makerere University (Kampala, Uganda) to conduct a randomized controlled trial of fractional-dose yellow fever vaccination in children 9–23 months of age. This trial will provide immunogenicity data needed to determine whether fractional-dose vaccination performs similarly to a full dose in the youngest age group eligible for yellow fever vaccination, further adding to the body of knowledge on the use of fractional-dose vaccination for outbreak response.

## Ebola Virus Disease

Human infection with Ebola virus causes hemorrhagic fever disease with a high case-fatality rate (*40*); sporadic outbreaks have been reported since 1976 (*41*). Within the genus *Ebolavirus *(family *Filoviridae*), 4 species are known to cause human disease: *Zaire ebolavirus *(ZEBOV), *Sudan ebolavirus*, *TaiïForest ebolavirus*, and *Bundibugyo ebolavirus*. Human-to-human transmission occurs through percutaneous or mucous membrane contact with blood or other body fluids of infected persons (*42**,**43*).

The Ebola outbreak in West Africa during 2014–2016 was the largest filovirus disease outbreak recorded and was caused by a ZEBOV strain. Over 24 months, this outbreak caused >28,000 suspected cases and >11,000 deaths in Guinea, Liberia, and Sierra Leone (*44*). Ebola vaccine delivery to at-risk populations during final stages of the outbreak was possible because of expedited vaccine development driven by the gravity of the public health emergency. During the outbreak, several clinical trials or investigational expanded access protocols used a single-dose, recombinant, replication-competent, vesicular stomatitis virus (VSV)−based vector encoding the ZEBOV glycoprotein (rVSV-ZEBOV). WHO and partners conducted a cluster-randomized ring vaccination trial in Guinea that showed 100% efficacy (95% CI 68.9%–100.0%) for randomized clusters of at-risk adults in rings, including contacts and contacts of contacts, of an infected person (*45*). Most adverse events were mild and self-limited; 2 serious adverse events (fever and anaphylaxis) were judged to be related to vaccination, and both case-patients recovered. This vaccine was offered to healthcare workers (HCWs) as part of clinical trials and as part of expanded access emergency ring vaccination for new clusters that arose in all 3 countries. The CDC Sierra Leone Trial to Introduce a Vaccine against Ebola (STRIVE) evaluated the largest safety sample in which no vaccine-associated adverse events were observed for nearly 8,000 participants (*46*). Although the vaccine has not yet been licensed, available evidence supports the efficacy and safety of the rVSV-ZEBOV vaccine in ring vaccination. Thus, rapid access to vaccine for at-risk groups is regarded by the public health community as a major adjunctive measure for consideration in future outbreak response.

CDC is engaged in assisting countries to incorporate vaccination delivery into emergency response plans. The development of guidelines and protocols for Ebola vaccination response will help ensure that activities are standardized, evidence-based, and well-coordinated with overall Ebola outbreak response efforts. Availability of a standard protocol approved for at-risk countries would facilitate evaluation of the vaccination response during an emergency.

CDC will support implementation research needed to inform policy decisions about use of an unlicensed Ebola vaccine, including additional regulatory approvals and requirements needed in the setting of expanded access, feasibility of vaccine introduction, potential interaction with ongoing immunization procedures or schedules, and vaccine acceptability and hesitancy in communities. Strategies to ensure adequate cold chain capacity for vaccine storage and transport to field sites will also need evaluation because the rVSV-ZEBOV vaccine requires storage at −60°C, which is not a standard capacity for national immunization programs in Africa. Stability data from the manufacturer (Merck & Co., Kenilworth, NJ, USA) suggest that single-dose vials (2 × 10^7^ PFU/mL) are stable for 2–8 days at 4°C (Merck & Co., pers. comm., 2017), which would improve the feasibility of using standard cold chain equipment to implement vaccination in remote areas.

Ring vaccination is the only vaccination strategy for Ebola with available effectiveness data to support its use (*45*). Additional response strategies include geographically targeted and HCW vaccination, but more research is needed to explore the effect of these strategies if used in the future. Geographically targeted vaccination may be most appropriate if areas of transmission are well-defined and densely populated. Vaccination strategies targeted geographically or focused on HCWs are also likely to be more feasible to implement quickly from fixed vaccination sites and would not require the high-quality contact tracing needed for ring vaccination. Postvaccination coverage evaluations would be used to assess success of the vaccination strategy. Preemptive vaccination for HCWs in high-risk countries is a strategy that might prevent another large-scale outbreak; data to support duration of effectiveness are needed to inform timing of revaccination and potential effects.

Licensure of the rVSV-ZEBOV vaccine is not expected until 2019, and additional candidate vaccines continue to be studied in clinical trials (*47**–**51*). During the prelicensure period, plans for emergency ring vaccination with the rVSV-ZEBOV vaccine should take into account new evidence and guidance to support use of alternate vaccine candidates or strategies. CDC has contributed to the development of preliminary guidance for implementation of a licensed Ebola vaccine as part of the Global Ebola Vaccine Implementation Team led by WHO (*52*).

## Conclusions

CDC emergency vaccine implementation activities enhance global health security by enabling more rapid containment of VPD outbreaks at their source. These activities have built in-country response capacity and have provided valuable evidence to inform future emergency vaccine delivery for the countries involved and globally for other countries at risk for VPD outbreaks. CDC has contributed to development and updating of guidelines that countries and partners use for response planning efforts; examples include an updated WHO position paper for cholera vaccines expected in 2017, an updated WHO position paper for typhoid vaccines expected in 2018, and the WHO Global Ebola Vaccine Implementation Team guidance document for Ebola vaccine implementation (*50*). Planning and evaluation of emergency vaccination present distinct challenges for predicting needs before an emergency, anticipating ways to expand vaccine availability during critical global shortages, and delivering and evaluating new products. The Ebola epidemic accelerated vaccine clinical trials and could set a precedent for rapid clinical development of countermeasures for future infectious disease outbreaks. Integration of vaccination with emergency response to VPD outbreaks will continue to augment global health security by reducing disease burden and mortality rates for vulnerable populations and by averting pathogen spread across international borders. Lessons learned from emergency vaccine implementation might inform response with new vaccines in the development pipeline, such as vaccines against Middle East respiratory syndrome coronavirus, Lassa virus, Marburg virus, and Zika virus, for which rapid response would also be required.
